# Adjuvants and MHCII modulate the immunogenicity of subdominant epitopes in *Plasmodium vivax* Duffy binding protein

**DOI:** 10.1016/j.isci.2025.113630

**Published:** 2025-09-23

**Authors:** Daniel Ferrer Vinals, Mohammad Rafiul Hoque, Opeyemi Ernest Oludada, Ethan B. Jansen, Catherine J. Mitran, Jhon R. Enterina, Matthew S. Macauley, Michael T. Hawkes, Stephanie K. Yanow

**Affiliations:** 1 School of Public Health, University of Alberta, Edmonton, AB T6G 2E1, Canada; 2 Department of Medical Microbiology and Immunology, University of Alberta, Edmonton, AB T6G 2E1, Canada; 3 Department of Chemistry, University of Alberta, Edmonton, AB T6G 2E1, Canada; 4 Department of Pediatrics, University of British Columbia, Vancouver, BC V6H 3V4, Canada

**Keywords:** Vaccinology, Immunology, Parasitology

## Abstract

Antibody responses to protein antigens are driven by epitope hierarchies that promote the immunogenicity of select epitopes. For antigenically variable pathogens, immune responses toward conserved epitopes are critical to elicit strain-transcending antibodies, yet these epitopes are often subdominant. Designing vaccines that focus immune responses to these epitopes requires an understanding of which components can overcome subdominance. Using the *Plasmodium vivax* Duffy binding protein with a defined subdominant region, we investigated how immunization strategies affect the immunogenicity of this region. We demonstrated that antigens with mutations in immunodominant epitopes, combined with the adjuvants GLA-SE or alum, overcame the subdominance of this region. However, these effects were largely negated by MHCII restriction. Further, robust antibody responses toward subdominant epitopes depend on boosting, which alters the epitope hierarchy and enables the recruitment of germinal center B cells specific to the subdominant epitopes. Our study provides valuable insights for designing subunit vaccines targeting subdominant epitopes.

## Highlights

• Adjuvants modulate antibody responses toward a subdominant region in PvDBPII
• Mutations in immunodominant epitopes further enhanced epitope-specific responses
• MHCII haplotype can restrict the immunogenicity of subdominant epitopes
• Boosting is required to achieve potent immunogenicity toward subdominant epitopes

## Graphical abstract

**Figure:**
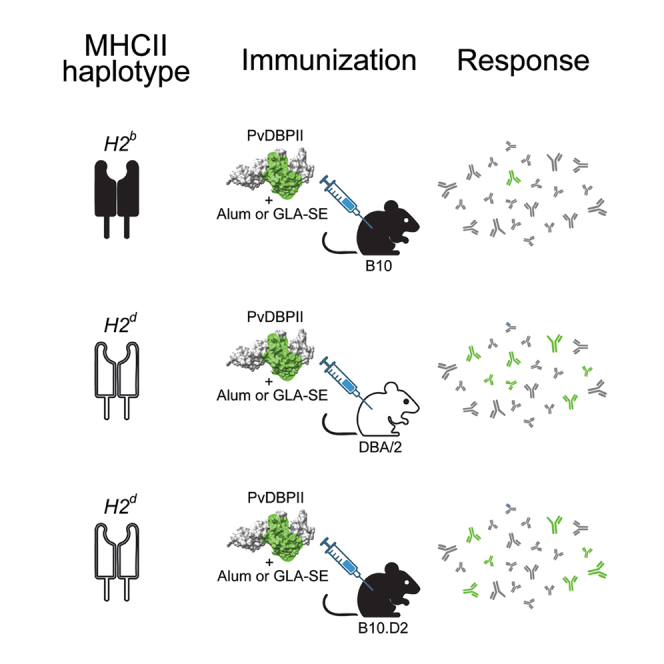

## Introduction

Vaccines are among the most effective tools to combat infectious diseases, although their efficacies against different pathogens vary significantly. The measles vaccine confers near 100% lifelong immunity, whereas influenza vaccines provide only limited seasonal protection against circulating strains.^1^^,^^2^ Understanding the barriers to achieving high vaccine efficacy is critical to alleviate the public health burden of infectious diseases such as influenza, HIV/AIDS, dengue, and malaria. Many vaccines target pathogen antigens to elicit protective, neutralizing antibodies. The challenge is that polyclonal responses yield quantitative differences in the production of antibodies toward different epitopes, and those that are immunodominant are not necessarily protective.^3^^,^^4^^,^^5^^,^^6^ For instance, immunization with the hemagglutinin antigen of influenza A elicits antibodies toward dominant polymorphic epitopes in the head structure of the hemagglutinin antigen at the expense of subdominant, conserved epitopes in the stalk region.^7^ This “epitope hierarchy” may reflect immune evasion strategies evolved by the pathogen to selectively engage host B cells that target non-neutralizing, strain-specific, and/or decoy epitopes while shielding more conserved subdominant epitopes.^8^

The design of vaccine antigens that focus antibody responses to desired epitopes is critical to overcome subdominance and requires a deeper understanding of the immunological factors that underpin dominance in the context of vaccination. The frequency of precursor B cells, the affinity and avidity of an epitope for its cognate B cell receptor (BCR), accessibility of an epitope to the BCR, and recruitment of T follicular helper cells all contribute to inter-clonal B cell competition that drives the production of epitope-specific antibodies.^9^^,^^10^ Vaccines can manipulate some of these mechanisms through antigen design and vaccine formulation. Antigens can be “resurfaced” to exclude or mask immunodominant epitopes (by mutation, truncation, or glycan masking, for example) to promote antigen binding to rarer B cells.^9^^,^^10^ Once activated, the expansion of these clones can be facilitated by adjuvants that stimulate antigen presentation to helper T cells and migration to germinal centers (GCs).^11^^,^^12^ While there are many different classes of adjuvants with distinct modes of action,^11^^,^^12^ several are reported to modulate epitope-specific responses, notably against antigens from HIV,^13^ *Staphylococcus aureus*,^14^ influenza,^15^ dengue virus,^16^ and *Plasmodium*.^17^ Certain adjuvants can clearly elicit antibodies with broader specificities, but it is not known whether they can overcome the subdominance of specific epitopes.

To better understand the effects of different adjuvants in driving epitope-specific responses, we used the *Plasmodium vivax* Duffy binding protein (PvDBP) as a model antigen. In pre-clinical mouse studies with recombinant PvDBP, the adjuvants alum or glucopyranosyl lipid adjuvant–stable emulsion (GLA-SE) promoted antibodies with greater neutralizing activity against polymorphic alleles of PvDBP, suggesting these adjuvants broaden antibody responses to conserved epitopes.^18^^,^^19^ Consistent with this, GLA-SE was associated with diversified sequences of the variable region in IgG and an increased number of linear peptides recognized by sera from these immunized mice.^19^ Notably, PvDBP contains a small (40 amino acids), well-defined subdomain (SD1) that is subdominant when mice are immunized with PvDBP adjuvanted with Titermax (Tmax).^20^ Thus, the subdominance of SD1 presents a valuable model to study how vaccine formulation can modulate the immunogenicity of subdominant epitopes. Here, we discovered that in certain strains of mice, SD1 subdominance was overcome when PvDBP was adjuvanted with alum or GLA-SE, and further enhanced with the genetic engineering of immunodominant epitopes. However, the increased immunogenicity of SD1 was MHCII-restricted, implying that the MHCII haplotype is a major driver of subdominance.

## Results

### Vaccine adjuvants enhance the immunogenicity of subdominant epitopes in *Plasmodium vivax* Duffy binding protein

**Figure 1:**
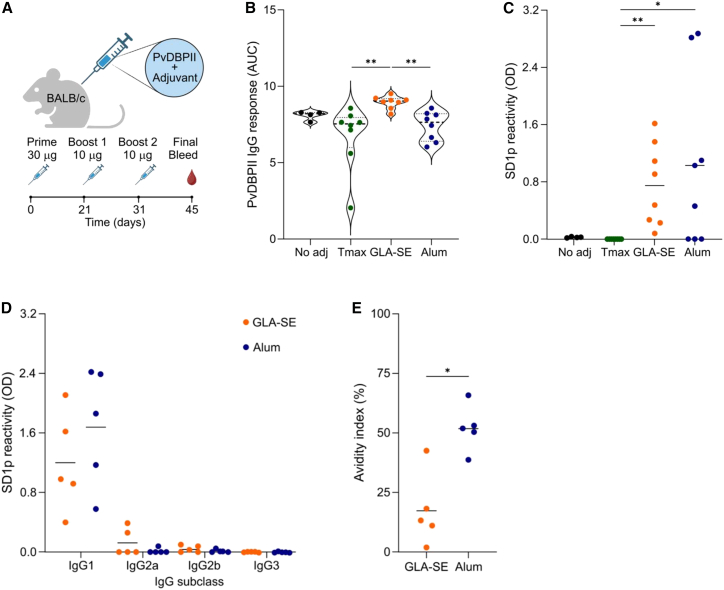
Adjuvants modulate antibody responses to SD1 within PvDBP vaccines (A) Experimental setup. BALB/c mice were immunized subcutaneously with a total dose of 50 μg/mouse of recombinant PvDBPII in the absence of adjuvant or with Tmax, GLA-SE, or alum. Mice received a prime and two boosts on days 21 and 31, and sera were collected on day 45. (B) Anti-PvDBPII IgG responses were determined by ELISA. Data are presented as the area under the curve (AUC) after subtracting the AUC from the adjuvant alone group. (C) Anti-SD1p IgG responses were determined by ELISA. OD_450nm_ values were determined from 1/500 diluted sera in immunized mice and background corrected using OD_450nm_ of sera from mice immunized with adjuvant alone. (D) Antibody isotypes of anti-SD1p IgG from the 5 highest responding mice in the alum and GLA-SE groups. (E) Avidity index of anti-SD1p IgG expressed as the antibody reactivity after treatment with 1M NaSCN compared to without treatment. Statistical analysis in B and C was performed using the Kruskal-Wallis test with *post hoc* Dunn’s multiple comparisons test, B (*p* = 0.0013), C (*p* = 0.0018). Analysis in D was performed by a two-way ANOVA with *post hoc* Tukey’s multiple comparison test (*P*_IgG subclass_ < 0.0001, *P*_adjuvant_ = 0.4899) and by the Mann-Whitney test in E (*p* = 0.0159). ∗*p* < 0.05, ∗∗*p* < 0.01. Dashed lines represent the median, and stippled lines represent the quartiles. Solid lines represent the mean.

Based on previous data that SD1 is subdominant when PvDBP is adjuvanted with Tmax,^20^ we compared its immunogenicity when PvDBP was formulated with GLA-SE or alum. Female BALB/c mice were immunized with recombinant PvDBP region II (PvDBPII) without adjuvant (*n* = 4) or adjuvanted with Tmax, GLA-SE, or alum (*n* = 8 per group). For each adjuvant, we also included a group immunized with adjuvant alone (*n* = 8), and seroreactivity values from these animals were subtracted from the corresponding groups immunized with PvDBPII. Mice received a prime followed by two homologous boosts on days 21 and 31 (Figure 1A). On day 45, all immunizations raised high antibody titers against PvDBPII, with the strongest antibody response observed with GLA-SE (Figure 1B). Despite similar titers against PvDBPII, reactivity to a 40 amino acid peptide that spans SD1 (“SD1p”)^21^ varied by adjuvant (Figure 1C). Since the reactivity to SD1p was generally low, we report the optical density (OD) values with sera diluted 1/500, which falls within the linear range of seroreactivity based on titration curves (Figure S1). Without adjuvant or with Tmax, the epitopes in SD1p were clearly subdominant, while both GLA-SE and alum increased the antibody responses to SD1p. These results were also observed in male BALB/c mice (Figure S2), suggesting that in both sexes, the choice of adjuvant can impact the immunogenicity of subdominant epitopes.

Given that GLA-SE stimulates strong Th1 responses whereas alum favors Th2 responses,^22^^,^^23^^,^^24^ we tested whether the anti-SD1p IgG elicited by different adjuvants was qualitatively different. The distribution of anti-SD1p IgG isotypes was similar between GLA-SE and alum, and the dominant IgG isotype was IgG1 (Figure 1D). When we compared the relative avidity, antibodies from the alum adjuvanted group had significantly greater avidity to SD1p (Figure 1E), suggesting alum promoted more extensive affinity maturation of antibodies to the epitopes in SD1.

**Figure 2:**
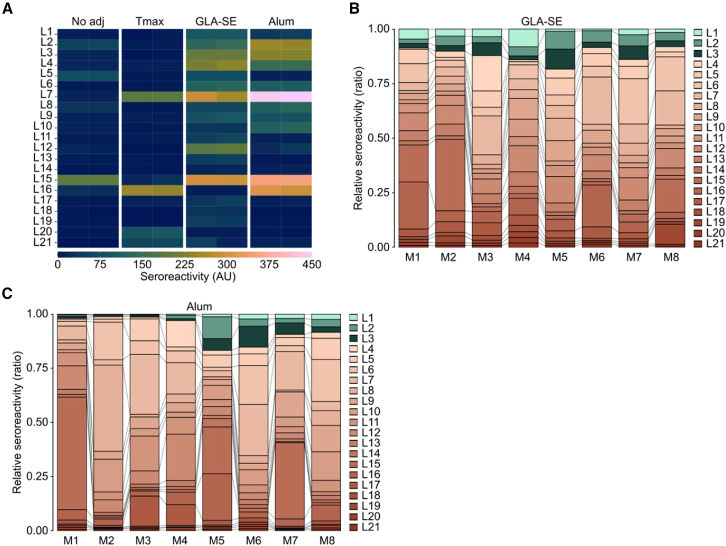
Effects of vaccine adjuvants on the epitope hierarchy of PvDBPII in BALB/c mice (A) Antibody reactivity of pooled sera from each group measured against 20-mer linear peptides spanning PvDBPII. OD values were converted to arbitrary units (AU) relative to an internal control. (B) Peptide-specific IgG responses of individual mice (M) after immunization with PvDBPII adjuvanted with GLA-SE. (C) Peptide-specific IgG responses of individual mice (M) after immunization with PvDBPII adjuvanted with alum. The peptides that encompass SD1p are colored in green.

Both GLA-SE and alum can increase the antibody reactivity to linear peptides within recombinant proteins.^14^^,^^16^^,^^19^ A key question emerging from our data is whether these adjuvants skewed the epitope hierarchy in PvDBPII specifically toward SD1p at the expense of other epitopes or simply increased the reactivity to linear epitopes generally. To answer this, we screened pooled sera from each group of immunized mice against a library of overlapping 20-mer linear peptides (L1 to L21) spanning the sequence of PvDBPII (Figure 2A). Sera from mice immunized without adjuvant or with Tmax recognized only a few linear peptides. Consistent with a previous study where GLA-SE increased the number of linear epitopes recognized in PvDBPII,^19^ we observed a more expansive antibody response to linear peptides in this library. Alum had a similar effect, although the specific pattern of reactive peptides was not identical to the GLA-SE group. To assess the variability in the epitope hierarchies of individual mice from the GLA-SE and alum groups, we analyzed sera from eight mice against the same library of peptides (Figures 2B and 2C). The seroreactivity to each peptide is expressed as a proportion of the total reactivity. Despite some variability among the mice and between the adjuvant groups, sera from most animals reacted to the peptides L1-L3 in SD1, L6-L7 in subdomain 2 (SD2), and L15-L16 in subdomain 3 (SD3).

### The immunogenicity of subdomain 1 can be further enhanced through mutations in *Plasmodium vivax* Duffy binding protein

**Figure 3:**
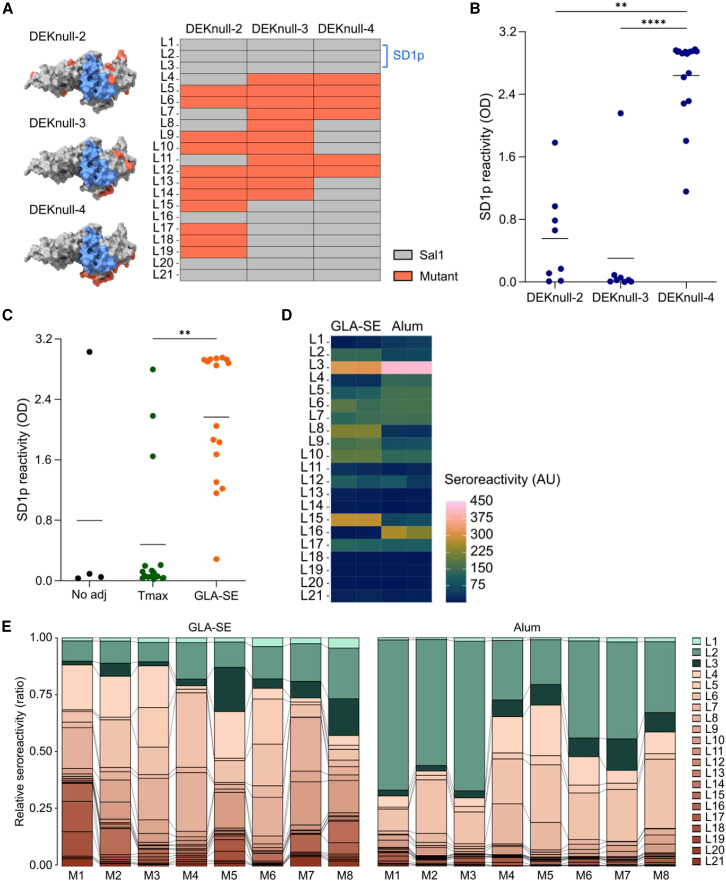
Mutations in PvDBPII can further increase the immunogenicity of SD1 (A) Schematic representation of antigens DEKnull-2, DEKnull-3, and DEKnull-4 with mutations highlighted in red mapped to the protein structure and the corresponding linear peptides L1 to L21. The conserved region SD1p is highlighted in blue. (B) Anti-SD1p IgG response from sera of BALB/c mice immunized with mutants DEKnull-2, DEKnull-3, or DEKnull-4 adjuvanted with alum. Sera were collected on day 45, and anti-SD1p IgG was measured by ELISA at a dilution of 1/500. (C) Anti-SD1p IgG response from sera of BALB/c mice immunized with DEKnull-4 only or DEKnull-4 adjuvanted with Tmax or GLA-SE. (D) Antibody reactivity of pooled sera from each group measured against 20-mer linear peptides spanning DEKnull-4. (E) Peptide-specific IgG responses of individual mice (M) after immunization with DEKnull-4 adjuvanted with GLA-SE or alum. OD values were converted to AU relative to an internal control. The peptides that encompass SD1p are colored in green. Statistical analysis in B and C was performed using Kruskal-Wallis test with *post hoc* Dunn’s multiple comparison test, B (*p* < 0.0001), C (*p* < 0.0001). ∗∗*p* < 0.01, ∗∗∗∗*p* < 0.0001. Solid lines on data plots represent the mean.

The peptide array data prompted us to ask whether mutations in the immunodominant peptides in SD2 and SD3 could further skew the epitope hierarchy to subdominant epitopes in SD1. To test this, we immunized mice with three recombinant PvDBP proteins (DEKnull-2 to 4)^25^ in which amino acids within SD2 and SD3 were mutated to serine, threonine, or alanine, including residues in L6, L7, or L15 (Figure 3A). Importantly, none of the mutations were in L1-L3 in SD1. We initially immunized mice with the DEKnull mutants adjuvanted with alum (Figure 3B) because this adjuvant elicited higher avidity antibodies to SD1p, and sera from this adjuvant group strongly recognized L7 and L15 in the peptide array (Figures 2A and 2C). DEKnull-2 elicited similar anti-SD1p responses to PvDBPII (compared to Figure 1C), whereas DEKnull-3 failed to elicit robust antibodies to SD1p even though antibody titers to the recombinant antigen were high (Figure S3A). We speculate that the mutations in this protein may have altered or compromised protein folding, which reduced the immunogenicity of SD1. In contrast, DEKnull-4 promoted a robust anti-SD1p response (Figures 3B and S3B). DEKnull-4 adjuvanted with GLA-SE also elicited a strong anti-SD1p response, while Tmax or no adjuvant increased the anti-SD1 antibodies in a few mice (Figure 3C).

To characterize the epitope hierarchy of antibodies elicited by vaccination with DEKnull-4 adjuvanted with GLA-SE or alum - the formulations that elicited the strongest antibodies to SD1p - we used the same library of linear peptides but substituted L4-7, L11, and L12 for peptides with the corresponding mutations in DEKnull-4. The most striking difference in the epitope hierarchy was the dominant reactivity of sera from both GLA-SE and alum adjuvanted groups against peptide L3 in SD1 (Figure 3D). Correspondingly, reactivity to the mutant L7 peptide was strongly reduced and no longer immunodominant. The sera of individual animals from the two groups confirmed our observations that SD1 is immunodominant with both adjuvants (Figure 3E). This response was most striking in the alum group, where the responses against L2-L3 dominated the reactivity in most of the animals (Figure 3E). Although we cannot exclude that specific conformational epitopes may be dominant under these immunization conditions, our findings support our earlier observations with PvDBPII that GLA-SE or alum increased antibody reactivity to more linear epitopes. This adjuvant effect, combined with antigen engineering, further promoted the immunogenicity of subdominant epitopes in SD1.

### Adjuvant effects on subdominant epitopes are strain-specific

**Figure 4:**
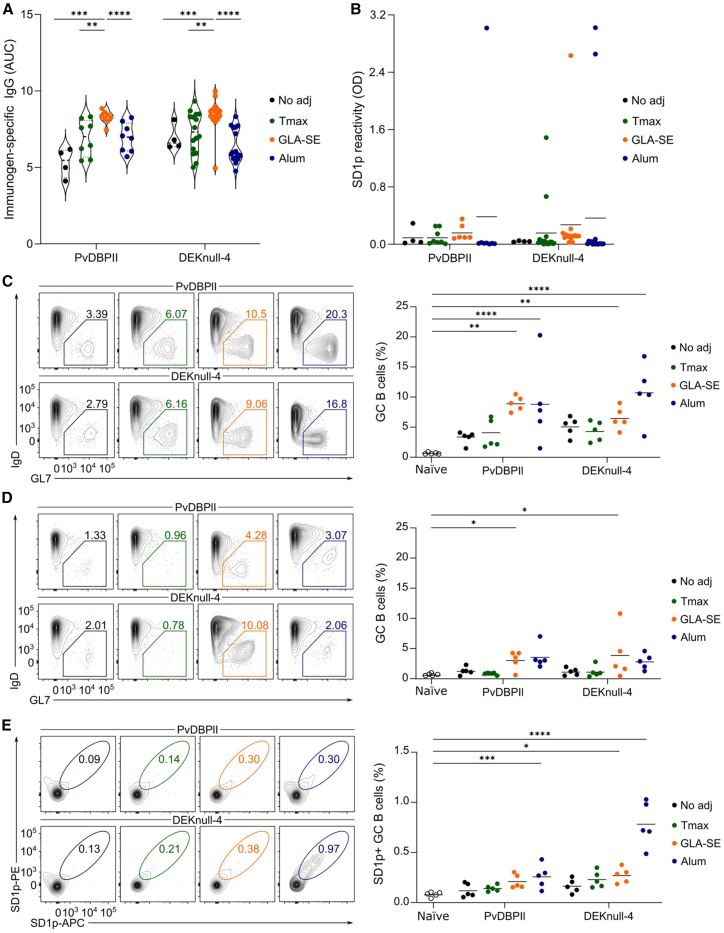

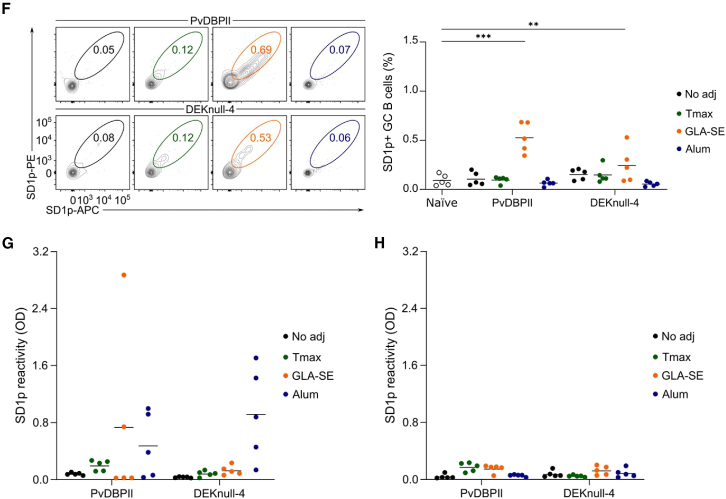
Adjuvant effects on subdominant epitopes are strain-specific (A) C57BL/6 mice were immunized with recombinant PvDBPII or DEKnull-4, administered alone or in combination with adjuvants. Immunogen-specific IgG responses were measured on day 45 post-immunization. Data are presented as the area under the curve (AUC) after subtracting the AUC from the adjuvant alone group. (B) IgG responses to SD1p on day 45, measured at 1/500 dilution. (C) Quantification of GC B cell responses to vaccination in BALB/c mice relative to naive mice. Left: representative flow cytometry gating of GC B cells (IgD-GL7+) from an individual mouse at day 14. Right: percentages of GC B cells stained from the draining lymph node. (D) Quantification of GC B cell responses to vaccination in C57BL/6 mice relative to naive mice. Left: representative flow cytometry gating of GC B cells (IgD-GL7+) from an individual mouse at day 14. Right: percentages of GC B cells stained from the draining lymph node. (E) Quantification of the SD1p-specific B cell response at day 14 in BALB/c mice relative to naive mice. Left: representative flow cytometry gating of the population of SD1p-specific GC B cells identified as double-positive (APC^+^PE^+^) using fluorescently labeled tetramers, SD1p-APC and SD1p-PE. Right: percentages of SD1p-specific GC B cells (relative to total B cells) stained from the draining lymph node. (F) Quantification of the SD1p-specific B cell response at day 14 in C57BL/6 (F) mice relative to naive mice. Left: representative flow cytometry gating of the population of SD1p-specific GC B cells identified as double-positive (APC^+^PE^+^) using fluorescently labeled tetramers, SD1p-APC and SD1p-PE. Right: percentages of SD1p-specific GC B cells (relative to total B cells) stained from the draining lymph node. (G) IgG responses in BALB/c mice against SD1p at day 14, measured at 1/500 dilution. (H) IgG responses in C57BL/6 mice against SD1p at day 14, measured at 1/500 dilution. Statistical analysis was performed using a two-way ANOVA with *post hoc* Tukey’s (A, B, G, and H) or Dunnett’s (C, D, E, and F) multiple comparison test, A (*P*_adjuvant_ = 0.0001; *P*_antigen_ = 0.5212), B (*P*_adjuvant_ = 0.5482; *P*_antigen_ = 0.8161), C (*P*_adjuvant_ = 0.0002; *P*_antigen_ = 0.1135), D (*P*_adjuvant_ = 0.0014; *P*_antigen_ = 0.7993), E (*P*_adjuvant_ < 0.0001; *P*_antigen_ = 0.0008, F (*P*_adjuvant_ < 0.0001; *P*_antigen_ = 0.3489), G (*P*_adjuvant_ = 0.0441; *P*_antigen_ = 0.6315) and H (*P*_adjuvant_ = 0.0141; *P*_antigen_ = 0.2671). ∗*p* < 0.05, ∗∗*p* < 0.01, ∗∗∗*p* < 0.001, ∗∗∗∗*p* < 0.0001. Dashed lines represent the median and stippled lines represent the quartiles. Solid lines on data plots represent the mean.

To extend our analyses to other mouse strains, we tested whether vaccine formulation had similar effects on SD1 epitopes in C57BL/6 mice. We immunized female C57BL/6 mice with either PvDBPII or DEKnull-4 antigens using the adjuvants Tmax, GLA-SE or alum and followed the same immunization regimen used for the BALB/c mice (Figure 1A). For each protein, we observed the highest immunogen-specific titers with GLA-SE (Figure 4A). The groups immunized with PvDBPII or DEKnull-4 without adjuvant also elicited antibody responses to their respective immunogens, but with lower titers compared to the adjuvanted groups. Surprisingly, neither PvDBPII nor DEKnull-4 elicited robust anti-SD1p IgG responses, even when adjuvanted with GLA-SE or alum (Figure 4B). These effects were specific to SD1p since the overall titers to PvDBPII or DEKnull-4 were similar between the two strains of mice (Figures 1B and 4A).

To understand the mechanisms underpinning the difference in SD1 immunogenicity in the two mouse strains, we analyzed early immunological responses on day 14 after the primary vaccination (Figure S4A). At this time point, IgG responses to the recombinant immunogen were detected in sera from both mouse strains and the different immunization groups (Figure S4B). In both mouse strains, DEKnull-4 elicited higher antibody responses compared to PvDBPII (Figure S4B), while the effect was more pronounced in BALB/c (*p* = 0.0001) than in C57BL/6 (*p* = 0.0265). Cells from the draining lymph nodes were harvested and gated for GC B cells (Figures 4C, 4D, and S5A). In the BALB/c mice immunized with either PvDBPII or DEKnull-4, both GLA-SE and alum promoted the expansion of total GC B cells compared to naive mice (Figure 4C), whereas in C57BL/6 mice, only GLA-SE promoted increased GC B cells (Figure 4D). To enumerate SD1p-specific GC B cells, we conjugated biotinylated SD1p to fluorescently labeled streptavidin, yielding SD1p-tetramer-APC and SD1p-tetramer-PE. We validated the specificity of both tetramer probes using FMO and non-immunized animals as controls (Figure S5B). Then, we stained and gated GC B cell populations labeled with both SD1p-tetramer-APC and SD1p-tetramer-PE probes (Figures 4E and 4F). In BALB/c mice, the alum groups showed expanded SD1p^+^ GC B cells for both immunogens. However, in GLA-SE adjuvanted groups, only the DEKnull-4 vaccine resulted in SD1p+ GC B cell responses (Figure 4E). On the other hand, in C57BL/6 mice, only the vaccines adjuvanted with GLA-SE had significantly greater SD1p^+^ GC B cells compared to naive animals (Figure 4F). The frequency of SD1p^+^ GC B cells did not correlate with the SD1p IgG responses on day 14, especially in the C57BL/6 mice (Figures 4G and 4H). These findings suggest that clonal selection within the GC after the prime or subsequent recall responses to the boosts contributed to the immunogenicity of the epitopes in SD1.

### MHCII haplotype modulates the subdominant subdomain 1 response

For antigen presenting B cells, the density of MHC-II/peptide complexes regulates their interaction with T cells to promote B cell expansion and differentiation to antibody-secreting cells.^26^^,^^27^ Important distinctions between BALB/c and C57BL/6 mice are the haplotype and number of MHCII genes. BALB/c are genetically characterized by two *H2*^*d*^ alleles (I-A and I-E) while C57BL/6 mice have one *H2*^*b*^ allele (I-A) and one null allele (I-E). We confirmed that the MHCII levels were significantly higher on SD1p-specific B cells from BALB/c compared to C57BL/6 mice (Figure S6A). MHCII levels on GC B cells are in part supported by globotriaosylceramide (Gb3), a glycosphingolipid that mediates the ranslocation of CD19 to the B cell receptor, activating the signaling cascade that facilitates the transition of GC B cells from the dark zone to the light zone and affinity maturation of B cells.^28^ Notably, when added exogenously as an adjuvant to a recombinant influenza hemagglutinin (rHA) vaccine in C57BL/6 mice, Gb3 broadened the diversity of antibody responses and strongly induced the production of antibodies to subdominant epitopes within the influenza HA stalk.^28^ We employed a similar approach to our PvDBP vaccine. We immunized C57BL/6 mice with recombinant PvDBPII and alum and compared the effect of Gb3 co-administration. Mice immunized with alum and Gb3 (no antigen) served as controls for background from the adjuvants (data not shown). In these experiments, mice were given a prime then one boost on day 21, with serum collected on days 14 and 30 (Figure S6B). Despite high antibody titers to PvDBPII (Figure S6C), Gb3 did not increase the immunogenicity of SD1p, even after the boost (Figure S6D).

**Figure 5:**
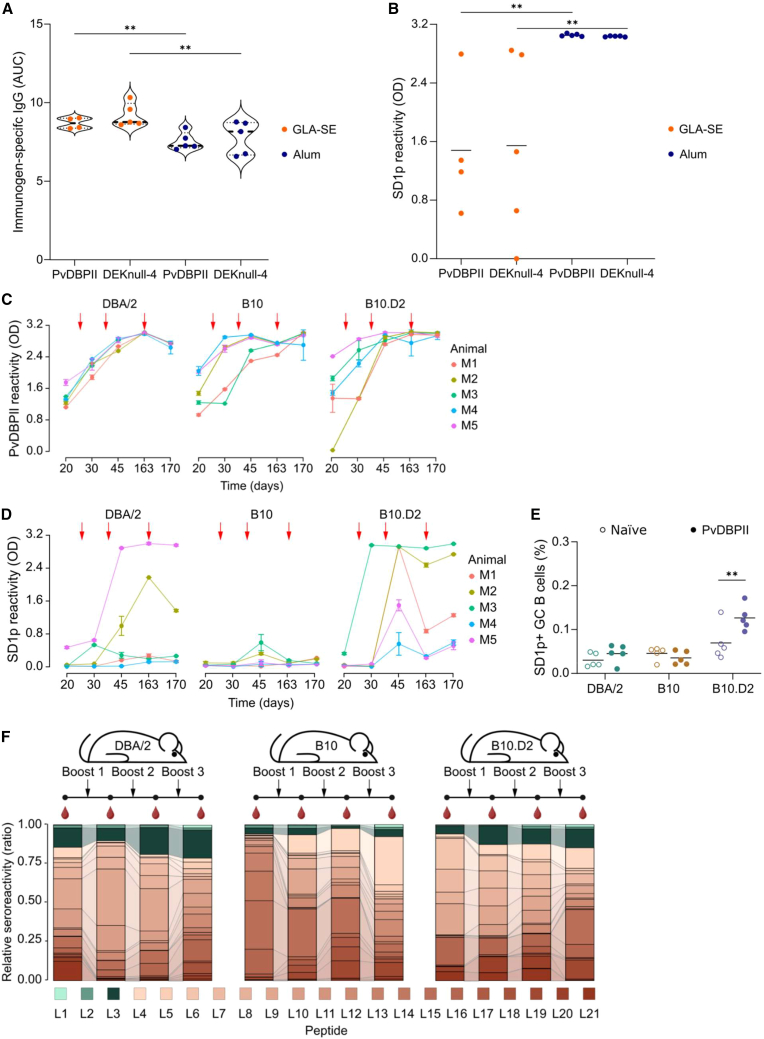
Strain-dependent immunogenicity of subdominant epitopes is largely modulated by MHCII haplotype (A) B10.D2 mice were immunized with PvDBPII or DEKnull-4 adjuvanted in GLA-SE or alum. Immunogen-specific IgG was measured on day 45 after prime and two boosts at a 1/2500 dilution. (B) IgG responses against SD1p measured at 1/500 dilution. (C) Time course of the IgG responses against PvDBPII elicited in the mouse strains DBA/2, B10, and B10.D2 immunized with PvDBPII and adjuvanted with alum. Red arrows indicate the homologous boosts given to animals on days 21, 31, and 163. Sera were diluted 1/2500. (D) Time course of the IgG responses against SD1p elicited in the mouse strains DBA/2, B10 and B10.D2 immunized with PvDBPII and adjuvanted with alum. Red arrows indicate the homologous boosts given to animals on days 21, 31, and 163. Sera were diluted 1/500. (E) Quantification of the SD1p-specific GC B cells stained from draining lymph nodes in DBA/2, B10 and B10.D2 mice on day 170. Data are the percentages of SD1p-specific GC B cells compared to naive animals. (F) Peptide-specific contributions in the IgG responses to PvDBPII immunizations adjuvanted with alum in the mouse strains DBA/2, B10, and B10.D2. Responses were measured in pooled sera at 1/500 dilution. Data are presented as relative seroreactivity. The peptides that encompass SD1p are colored in green. For A and B, significance was calculated using a two-way ANOVA with *post hoc* Tukey’s multiple comparison test, A (*P*_adjuvant_ = 0.0014; *P*_antigen_ = 0.2838), B (*P*_adjuvant_ = 0.0004; *P*_antigen_ = 0.9546). For E, significance was calculated by a two-way ANOVA with Šídák’s multiple comparison test (*P*_strain_ < 0.0001; *P*_antigen_ = 0.0322). ∗∗*p* < 0.01. Dashed lines represent the median, and stippled lines represent the quartiles. Solid lines on data plots represent the mean.

We next tested whether the haplotype of the MHCII genes could be a determinant in SD1 suppression. We tested our vaccines in a third mouse strain, B10.D2, that also has the *H2*^*d*^ alleles of MHCII, such as BALB/c. On day 45 after prime and two homologous boosts (Figure 1A), high antibody titers were elicited against both PvDBPII and DEKnull-4 when adjuvanted with either GLA-SE or alum (Figure 5A). Strikingly, antibodies to SD1p were also high (Figure 5B). With either immunogen, alum mounted a more robust antibody response to SD1p compared with GLA-SE. To confirm that the MHCII haplotype was the primary driver of SD1 immunogenicity, we directly compared the B10.D2 with its parental strains, B10 and DBA/2. We immunized mice with PvDBPII and alum and followed the kinetics of the anti-SD1p IgG. To extend the vaccination regimen further, we gave mice a delayed third boost on day 163 and collected serum prior to this boost and 7 days later (day 170). All mice had high titers of anti-PvDBPII antibodies that increased with subsequent boosts (Figure 5C). For SD1p, after the primary immunization (day 20) only one mouse from each of the DBA/2 and B10.D2 groups had any SD1p antibodies and the B10 mice were not responsive (Figure 5D). After one boost (day 30), the anti-SD1p antibody titers further increased in both the DBA/2 mice and B10.D2 mice but B10 mice showed no improvement in anti-SD1p antibody levels. Finally, after two boosts (day 45), a second DBA/2 mouse and all five B10.D2 mice were responsive. In the B10 group, two mice had very low SD1p antibodies, but these responses were not boosted further. After the third boost, there were no increases in SD1p antibodies in the DBA/2 mice, while all five B10.D2 mice showed boosted SD1p levels. None of the B10 mice had SD1p antibodies on day 163 or after the third boost, consistent with the subdominance of SD1 associated with the *H2*^*b*^ haplotype. For all groups, we harvested draining lymph nodes on day 170 (day 7 after the third boost) and enumerated the SD1p^+^ GC B cells (Figure 5E). Consistent with the antibody data, we observed a significant increase in the recruitment of SD1p^+^ B cells into the GC in the B10.D2 mice compared to naive animals.

In our previous experiments with BALB/c mice, we observed that the adjuvant altered the epitope hierarchy of PvDBP (Figure 2). To characterize how the MHCII haplotype contributes to changes in the epitope hierarchy, we compared the antibody reactivity following the prime and three boosts in pooled sera from the DBA/2, B10, and B10.D2 mice immunized with PvDBPII and alum (Figure 5F). We expressed the reactivity to each peptide relative to the total reactivity to all 21 peptides in the library. In the DBA/2 mice, reactivity to the L3 peptide in SD1p represented a sizable fraction of the total seroreactivity and increased after the second boost. This contradicts the low reactivity to SD1p after the prime (Figure 5D, day 20) and may reflect a bias from the sera of the one mouse in the pool with SD1p antibodies or differences in the reactivity to the shorter peptides in the library compared to the single continuous SD1p peptide. Over the course of immunization, we noted reduced reactivity to peptides in SD2 and increased reactivity to certain peptides in SD3. The B10.D2 mice, which have the same MHCII genes as the DBA/2 strain, also had strong antibodies to peptide L3, and these were expanded after the first and second boosts, then remained relatively stable after the third boost. The rest of the hierarchy followed a pattern similar to the DBA/2 mice. As expected, the profile in B10 contrasts with both the DBA/2 and B10.D2 mice with the clear suppression of epitopes in SD1p, especially after the second boost. In this strain, the epitope hierarchy is skewed toward peptides from SD3 with some expansion of reactivity to peptides in SD2 after boost 1. While immunization in all three strains elicited dynamic changes to the epitope hierarchy overall, the immunogenicity of the subdominant epitopes in SD1p was distinctly modulated by the MHCII haplotype.

## Discussion

The current study used PvDBP as a model antigen to examine how the immunogenicity of subdominant epitopes can be modulated within the context of a human malaria vaccine. PvDBP is the leading vaccine candidate against *P. vivax,* and two vaccines based on the Sal1 allele, adjuvanted either with GLA-SE or Matrix M showed promising results.^29^^,^^30^ Both vaccines elicited antibodies with strain-transcending neutralizing activity against variants of PvDBP, measured either *in vitro*^30^ or following challenge with a heterologous parasite strain expressing the PvW1 allele of PvDBP.^29^ Vaccine formulation was based upon earlier pre-clinical studies that selected adjuvants diversified the antibody repertoire and correlated with inhibitory activity against diverse PvDBP alleles.^19^ In our study, we observed that GLA-SE and alum (but not Tmax) broadened the PvDBP antibody profile to encompass subdominant epitopes within SD1. These findings are consistent with the data for PvDBP and previous observations that adjuvants can shape the epitope hierarchy in other vaccine antigens. Among the early studies on recombinant malaria vaccines, Hui et al. (1991) examined the immunogenicity of gp195 (PfMSP1) formulated with 6 different adjuvants.^17^ Distinct patterns of reactivity to polypeptides were observed, suggesting the adjuvants modulated the immunogenicity of B cell epitopes within the recombinant immunogen. Later studies reported that adjuvants diversified the epitope hierarchies of candidate vaccines against other pathogens, including influenza,^15^ tick-borne encephalitis virus,^31^ *Staphylococcus aur*eus,^14^ and dengue virus.^16^ Despite these observations, the mechanisms by which adjuvants can change the epitope hierarchy are not known. In our study, the adsorption of PvDBP to alum may have induced conformational changes that exposed linear epitopes^32^ or prolonged the bioavailability of the antigen through a depot effect, although these may not be the primary immunostimulatory mechanisms for alum.^33^^,^^34^^,^^35^ The diversification of epitopes by GLA-SE may be mediated by enhanced APC activation and antigen uptake,^36^^,^^37^^,^^38^ altered antigen processing and presentation pathways, and modulation of T cell help,^39^ potentially favoring the recognition of a broader range of epitopes.^40^

While GLA-SE and alum promoted broad immunogenicity of linear epitopes in PvDBPII, the genetically engineered DEKnull-4 further focused the antibody response to epitopes in SD1. DEKnull-4 has several mutations including sequences within SD2 that were immunodominant when PvDBPII was adjuvanted with either of the three adjuvants. Following immunization with DEKnull-4, the antibody response was skewed to L2 and L3 in SD1. We do not know whether the mechanism underpinning this epitope re-focusing is structural or immunological. DEKnull-4 is mutated in sequences required for the dimerization of PvDBP,^25^^,^^41^ which could alter the protein structure and physically expose SD1, increasing B cell recognition. Consistent with this, molecular dynamics simulation predicted that SD1 is more structurally stable in DEKnull-4 compared to PvDBPII (data not shown). Alternatively, the mutations in DEKnull-4 may favor activation and expansion of rarer B cells that recognize epitopes in SD1. This mechanism is akin to antigen resurfacing or glycan masking to suppress immunodominance within a competitive environment.^42^^,^^43^^,^^44^^,^^45^

Ultimately, the adjuvant and protein engineering strategies that effectively promoted the immunogenicity of epitopes in SD1 were restricted by the mouse MHCII haplotype. Mice with the MHCII *H2*^*b*^ haplotype (C57BL/6 or B10) generally failed to promote the immunogenicity of SD1, even with the optimal antigen and adjuvant formulations observed in the *H2*^*d*^ mice. Given that an influenza HA vaccine adjuvanted with Gb3 increased the immunogenicity of subdominant epitopes within the rHA stalk in *H2*^*b*^ mice^28^ we expected Gb3 to overcome the subdominance of SD1, but this effect was not observed, perhaps due to the much smaller size of the SD1p peptide. When we examined the early B cell responses in C57BL/6 mice, GLA-SE promoted the recruitment of SD1p^+^ B cells into the GC after the prime, but this was not associated with SD1p-specific IgG after prime or homologous boosts. None of the other immunization conditions promoted SD1p^+^ GC B cells after the prime, nor after a delayed third boost. Given that MHCII expression is lower in C57BL/6 mice compared to BALB/c, this may reflect a more competitive environment for rarer B cells that bind to subdominant epitopes. Competition among B cell clones can either impede their entry into the GC (the “pre-GC checkpoint”^46^) or block their positive selection within the GC, resulting in failure to form plasmablasts.^47^^,^^48^

An outstanding question is how these MHCII alleles can modulate subdominant responses. Each MHCII allele has a greater affinity to specific T cell epitopes, but how this can drive competition among epitope-specific B cells is not known. One mechanism could be through T-B reciprocity.^49^ T epitopes within or adjacent to SD1 could be shielded by the BCR complex from proteolytic digestion in the endosome when antigen is taken up by receptor-mediated endocytosis on each B cell, and these peptides could be preferentially loaded onto MHCII molecules to engage helper T cells and expand these B cell clones. Differences in T epitope bias (peptide binding affinity to each MHC II allele) between the *H2*^*b*^ and *H2*^*d*^ alleles could direct T cell help to SD1-responsive B cells in one strain but not the other.^50^^,^^51^ Alternatively, it is possible that the BCR-SD1 complex masks this T epitope, preventing it from being loaded onto MHCII in the *H2*^*b*^ mice, which could limit access to T cell help. However, MHCII haplotype alone is likely insufficient to explain subdominance, and other factors, such as the density of peptide loaded MHCII (due to the affinity of the different T epitopes for the MHCII alleles) could also contribute to the stringency of anti-SD1 responses.^27^ We observed the most robust responses to SD1 in B10.D2 mice, which carry the *H2*^*d*^ haplotype, yet this response was greater than the BALB/c and DBA/2 mice, which carry the same alleles. Further, the epitope hierarchies were not identical among strains with the same haplotype. This may be due to the variability of antibody responses within the pooled sera or reflect a role for other genes that distinguish each of the mouse strains, including immunoglobulin variable and J region repertoires. It should be noted that the comparison between B10.D2 and its parental strain B10 is the best controlled for MHC effect because these strains are congenic, that is, genetically identical except for the difference in MHC. In contrast, the difference between BALB/c (or DBA/2) and C57BL/6 is not limited to MHC but also includes other immunological differences. For example, BALB/c responses tend to be more skewed to Th2 cytokine profiles than C57BL/6, which tend to be more skewed to Th1 cytokine profiles. The cytokine profiles can affect the IgG subclasses of the antibody response. Thus, it is notable that the best response to SD1 was in B10.D2, which shares its non-MHC genes with B10, which had the lowest responses. This comparison implies that the main genetic effect can be attributed to the MHC difference and probably the difference in helper epitopes presented by I-A^*b*^ vs. I-A^*d*^ and I-E^*d*^.

A key observation in our experiments is that even in mice with the *H2*^*d*^ haplotype, boosting (usually twice) with homologous antigen was required to elicit robust antibodies to SD1. These results suggest that the recall response is critical to increase the production of anti-SD1 IgG. Notably, this recall response failed in B10 mice with the *H2*^*b*^ haplotype. These findings could reflect the poor formation of memory B cells specific to SD1, which prevents recruitment to new GCs following second or third immunizations. Another mechanism is through antibody feedback, which is known to alter the epitope hierarchy following prime and boost immunizations.^52^^,^^53^^,^^54^^,^^55^^,^^56^^,^^57^ In response to boosting, antibodies to immunodominant epitopes that are elicited after the prime and persist in the serum can bind to the epitopes in the vaccine, preventing memory B cells from interacting with the epitope, resulting in the expansion of B cells to subdominant epitopes. This was elegantly demonstrated with the *P. falciparum* CSP protein.^57^ Antibody titers against the immunodominant NANP repeat region plateaued after two immunizations, while B cell responses to subdominant epitopes in the C-terminus increased, promoting the diversification of the humoral response. Adjuvants may contribute to the antibody feedback mechanism by promoting antigen presentation and increasing antibody titers with each immunization, in turn driving these changes in epitope hierarchies.

The epitope-specific responses reported here have important implications for vaccine design. For *Plasmodium* and other genetically diverse pathogens, antibody-mediated immunity is hampered by immunodominant responses that are strain-specific or fail to neutralize the pathogen.^43^^,^^56^^,^^58^^,^^59^ Understanding how the choice of adjuvants and antigen design can direct antibody responses toward desired subdominant epitopes could inform new vaccine strategies.^24^ However, probing the mechanisms of MHCII restriction on epitope hierarchies will be key to achieving robust and predictable epitope-specific responses in human populations.

### Limitations of the study

Although we inferred mechanisms of altered epitope hierarchies from peptide mapping and antibody profiling, we were unable to directly measure the effects of adjuvants or the engineered mutations on the structure of the antigen. Our characterization of the epitope hierarchies was also limited to linear epitopes. Our study included only select MHCII haplotypes in inbred mice. Finally, our work was conducted in mouse models, and the extent to which these adjuvant and haplotype-dependent effects predict vaccine performance in humans remains to be tested.

## Resource availability

### Lead contact

Requests for further information and resources should be directed to and will be fulfilled by the lead contact, Stephanie K. Yanow (yanow@ualberta.ca).

### Materials availability

This study did not generate new unique reagents.

### Data and code availability

• All data reported in this article will be shared by the lead contact upon request.
• All computational code is available at http://github.com/Yanow-lab/Vinals-Hoque-et-al.
• Any additional information required to reanalyze the data reported in this article is available from the lead contact upon request.

## Acknowledgments

We thank Payton LeBlanc for assistance with animal immunizations. We thank Francis Ntumngia and John H. Adams (University of South Florida) for providing the plasmid constructs for the expression of the recombinant proteins, the DEKnull-2 recombinant antigen, and the 3D10 monoclonal antibody. We thank Michael Good and Jay Berzofsky for their valuable feedback and comments on the article. We also thank the staff of the Health Sciences Laboratory Animal Services at the University of Alberta for their assistance with animal housing and maintenance. Experiments were performed at the University of Alberta Faculty of Medicine & Dentistry Flow Cytometry Facility, RRID:SCR_019195, which receives financial support from the Faculty of Medicine & Dentistry and Canada Foundation for Innovation (CFI) awards to contributing investigators. This research was funded by the National Institute of Allergy and Infectious Diseases of the National Institutes of Health (R01AI150944). The content is solely the responsibility of the authors and does not necessarily represent the official views of the National Institutes of Health. We acknowledge funding from a project grant from the Canadian Institutes of Health Research (10.13039/501100000024CIHR_IRSC Funding Reference Number 168944). M.R.H. was supported by the Public Health doctoral scholarship from the School of Public Health at the University of Alberta. The funders played no role in study design, data collection, analysis and interpretation of data, or the writing of this article.

## Author contributions

D.F.V., M.R.H., and S.K.Y. designed the study and experiments. D.F.V. performed the peptide library screening, M.R.H. expressed and purified the recombinant proteins, and executed the animal experiments. M.R.H., D.F.V., and C.J.M. performed the serology analysis. J.R.E. designed the flow panel with guidance from M.S.M.; M.R.H., E.B.J., and D.F.V. performed the flow cytometry. O.E.O. analyzed flow cytometry data. M.T.H. assisted with the statistical analysis. S.K.Y., D.F.V., and M.R.H. wrote the article. S.K.Y. supervised the study. All authors read and approved the final article.

## Declaration of interests

All authors declare no financial or non-financial competing interests.

## STAR★Methods

### Key resources table

**Table:** 

REAGENT or RESOURCE	SOURCE	IDENTIFIER
**Antibodies**		

Rat anti-mouse B220 (clone:RA3-6B2)	BioLegend	cat# 103241; RRID: AB_11204069
Rat anti-mouse CD19 (clone: 6D5)	BioLegend	cat# 115555; RRID: AB_2565970
TruStain FcX™ PLUS (anti-mouse CD16/32)	BioLegend	cat# 156604; RRID: AB_2783137
Rat anti-mouse IgD (clone: 11–26c.2a)	BD Biosciences	cat# 563618; RRID: AB_2738322
Rat anti-mouse/human GL7 (clone: GL7)	BioLegend	cat# 144620; RRID: AB_2800676
Rat anti-mouse I-A/I-E (clone: M5/114.15.2)	BioLegend	cat# 107639; RRID: AB_2565894
Goat anti-mouse IgG (H + L)-HRP conjugate	Bio-Rad	cat# 170-6516; RRID: AB_2921252
Goat anti-mouse IgG 1 HRP	Invitrogen	cat# PA1-74421; RRID: AB_10988195
Goat anti-mouse IgG2a HRP	Invitrogen	cat# A10685; RRID: AB_2534065
Goat anti-mouse IgG2b HRP	Invitrogen	cat# M32407; RRID: AB_10563452
Goat anti-mouse IgG3 HRP	Abcam	cat# AB97260; RRID: AB_10680425
Goat anti-mouse IgG Alexa Fluor 647	Invitrogen	cat# A21237; RRID: AB_2535806
Mouse anti-PvDBPII monoclonal antibody (3D10)	Gift from Dr. John Adams, U of Florida	N/A

**Chemicals, peptides, and recombinant proteins**		

7-AAD	BioLegend	cat# 420403
APC Streptavidin	BioLegend	cat# 405207
PE-Streptavidin	BioLegend	cat# 405204
Gb3 Globotriaosylceramide	Cayman Chemicals	cat# 24876
PvDBP (Salvador-1)	Prepared in the lab	N/A
PvDBP (DEKnull-2)	Prepared in the lab	N/A
PvDBP (DEKnull-3)	Prepared in the lab	N/A
PvDBP (DEKnull-4)	Prepared in the lab	N/A
Alhydrogel (aluminium hydroxide)	InvivoGen	cat# vac-alu-250
GLA-SE	Advanced Health Institute (AAHI)	cat# EM082
TiterMax® Gold Adjuvant	Millipore Sigma	cat# T2684
3,3′,5,5′-Tetramethylbenzidine(TMB) Substrate for ELISA	Millipore Sigma	cat# T0440
96-Well Plates	Thermo Fisher	cat# 439454
Bovine serum albumin (BSA)	Millipore Sigma	cat# A7906
ISSAIINHAFLQNTVMKNCN	Pepmic Co., Ltd	L1
MKNCNYKRKRRERDWDCNTK	Pepmic Co., Ltd	L2
DCNTKKDVCIPDRRYQLCMK	Pepmic Co., Ltd	L3
QLCMKELTNLVNNTDTNFHR	Pepmic Co., Ltd	L4
TNFHRDITFRKLYLKRKLIY	Pepmic Co., Ltd	L5
RKLIYDAAVEGDLLLKLNNY	Pepmic Co., Ltd	L6
KLNNYRYNKDFCKDIRWSLG	Pepmic Co., Ltd	L7
RWSLGDFGDIIMGTDMEGIG	Pepmic Co., Ltd	L8
MEGIGYSKVVENNLRSIFGT	Pepmic Co., Ltd	L9
SIFGTDEKAQQRRKQWWNES	Pepmic Co., Ltd	L10
WWNESKAQIWTAMMYSVKKR	Pepmic Co., Ltd	L11
SVKKRLKGNFIWICKLNVAV	Pepmic Co., Ltd	L12
LNVAVNIEPQIYRWIREWGR	Pepmic Co., Ltd	L13
REWGRDYVSELPTEVQKLKE	Pepmic Co., Ltd	L14
QKLKEKCDGKINYTDKKVCK	Pepmic Co., Ltd	L15
KKVCKVPPCQNACKSYDQWI	Pepmic Co., Ltd	L16
YDQWITRKKNQWDVLSNKFI	Pepmic Co., Ltd	L17
SNKFISVKNAEKVQTAGIVT	Pepmic Co., Ltd	L18
AGIVTPYDILKQELDEFNEV	Pepmic Co., Ltd	L19
EFNEVAFENEINKRDGAYIE	Pepmic Co., Ltd	L20
GAYIELCVCSVEEAKKNTQE	Pepmic Co., Ltd	L21
QLCMKALTNLVNNTDTNFHR	Pepmic Co., Ltd	L4_DEKnull-4
TNFHRDITARKAYLAAKLTA	Pepmic Co., Ltd	L5_DEKnull-4
AKLTADAASEGDLLLKLAAT	Pepmic Co., Ltd	L6_DEKnull-4
KLAATATSAATCKDIRWSLG	Pepmic Co., Ltd	L7_DEKnull-4
WWNESKATIWTAMMASATAS	Pepmic Co., Ltd	L11_DEKnull-4
SATASAATSAATAAKLNVAV	Pepmic Co., Ltd	L12_DEKnull-4
Biotin-Ahx- ISSAIINHAFLQNTV MKNCNYKRKRRERDWDCN TKKDVCIPDRRYQLCMK	Pepmic Co., Ltd	SD1-Biotin

### Experimental model and study participant details

#### Mouse strains

All mice used in this study were 6–8 weeks old. Male and/or female BALB/c (stock no.:028) and female C57BL/6 (stock no.:027) mice were purchased from Charles River, Canada and B10.D2 (stock no.:000463), B10 (stock no.:000666), and DBA/2 (stock no.:000671) female mice were purchased from The Jackson Laboratory, USA. The experiments described in this study were approved by the Health Sciences Animal Care and Use Committee of the University of Alberta (AUP00002124). All mice were housed at Health Sciences Laboratory Animal Services at the University of Alberta, in individually ventilated cages maintained at 20°C–24°C and 40–70% humidity, with a 12-h light/12-h dark cycle and *ad libitum* access to food and water.

### Method details

#### Recombinant protein expression

The plasmids encoding PvDBPII (Salvador I strain), DEKnull-3, and DEKnull-4 and the DEKnull-2 protein were a generous gift from Dr. John Adams, University of South Florida. Proteins were expressed in the *E. coli* system as per standard protocol and confirmed by SDS-PAGE and western blot with 3D10 (Figure S7). Briefly, plasmid DNA was transformed into BL21 (DE3) competent cells. IPTG (Isopropyl-β-*d*-thiogalactopyranoside) (1.0 mM) was used to induce the recombinant protein expression for 3 h at 30°C. Protein solubilization, purification, and refolding were performed as described elsewhere.^60^ The refolded proteins were eluted by ion exchange chromatography using 1 M NaCl. Finally, buffer exchange was performed with PBS.

#### SDS-PAGE and western blot analysis

Refolded recombinant proteins were separated by a 4–15% gradient SDS-PAGE and stained with 0.25% Coomassie brilliant blue. Briefly, 5 μg refolded proteins were incubated with 10 mM dithiothreitol (DTT, reduced condition), incubated at 37°C for 1 h followed by adding 2X loading dye containing reducing agent 2-mercaptoethanol; 5 μg protein without DTT (non-reduced condition) and added 2X loading dye without reducing agent. Samples were heated at 95°C for 4 min.

For western blot analysis, proteins were electro-transferred to a 0.45 μm nitrocellulose membrane. Electrophoresis was performed using a wet transfer system containing transfer buffer composed of 25 mM Tris and 192 mM glycine, with 20% methanol (w/v). A constant 100 V current was applied for 1 h. The membrane was then incubated overnight at 4°C in blocking buffer consisting of 5% skim milk in PBS. The blot was then treated with 3D10 antibody (1:2500) diluted in 5% skim milk in a 0.1% PBS-Tween 20 solution at room temperature (RT) for 1.5 h, followed by incubation with secondary goat anti-mouse Alexa Fluor 647 antibody (1:5000) for 20 min at RT. The blot was imaged using a ChemiDoc™ MP imaging system.

#### Animal immunizations

Mice were immunized subcutaneously with 30 μg of PvDBPII (*n* = 8), DEKnull-2 (*n* = 8), DEKnull-3 (*n* = 8), DEKnull-4 (*n* = 16), either alone (n = 4) or in combination with 1:1 (v/v) of one of the adjuvants: Tmax, GLA-SE, or Alum. On days 21 and 31, mice were boosted with the immunogen (10 μg/mouse) either alone or in combination with the corresponding adjuvant, and the final sera samples were collected on day 45 by cardiac puncture. In certain experiments, a fourth dose of vaccine (10 μg/mouse; *n* = 5 per group) was administered on day 163, and the final sera were collected on day 170.

Prior to cardiac puncture, mice were anesthesized using 4% isoflurane with 1000 mL/min oxygen flow. The percent increment of isoflurane was slowly increased, starting with 0.5% and increasing by 0.5% increments every few breaths to a maximum of 4%, providing a smooth induction with minimal change in breath holding. The deep surgical plane anesthesia was confirmed by checking the toe pinch reflex on all four paws. Once deep anesthesia was achieved, blood was withdrawn from the heart. For experiments that required collection of lymph nodes, mice were euthanized using CO_2_ euthanasia (maintaining a 2.2 L/min flow rate of CO_2_). Then, blood and lymph nodes were collected following complete cessation of respiration.

The Gb3-containing vaccine formulation was prepared as described elsewhere.^28^ Briefly, 10 μL (5 mg/mL) of Gb3 stock (dissolved in dimethyl sulfoxide) solution was mixed with PvDBPII containing saline solution. This mixture was emulsified using 10% Tween 80 for 30 min at room temperature. Next, each mouse received this lipid-based formulation in combination with 1:1 (v/v) of alum. An equivalent amount of PvDBPII was used for prime (30 μg/mouse) and boost (10 μg/mouse) in the Gb3 immunization scheme.

#### Enzyme-linked immunosorbent assay (ELISA)

To measure the antigen-specific reactivity of sera samples, we performed indirect ELISAs by coating 96-well plates with antigen diluted in 1X PBS, incubated overnight at 4°C. Recombinant protein antigens were coated at 0.5 μg/mL and synthetic peptides were coated at 1.0 μg/mL. After 1 h blocking with 4% BSA at 37°C, plates were washed once with 1X PBST (0.1% Tween 20). Primary antibody samples were diluted in 2% BSA, added to wells, and incubated for 1 h at room temperature (RT). Plates were washed four times with 1X PBST, and 100 μL of horseradish peroxidase (HRP)-conjugated goat anti-mouse IgG (1/3000), IgG1 (1/8000), IgG2a (1/1000), IgG2b (1/2000), and IgG3 (1/5000) secondary antibody was added to each well. After incubation for 1 h at RT, the plate was washed four times with 1X PBST, and 100 μL of ELISA substrate TMB was added to each well. After incubation at RT for 30 min, the reaction was stopped by adding an equal volume of H_2_SO_4_ (0.5 N) to each well, and the optical density (OD) of individual wells was read at 450 nm. All samples were run in duplicate and the mean OD for each antigen alone plus secondary was subtracted from the OD of each sample. The area under the curve (AUC) was calculated in Prism by fitting a sigmoidal curve, with the log dilution on the x axis and the corresponding OD value on the y axis.

To test the antibody avidity, 100 μL of 1 M NaSCN was added and incubated for 10 min at RT following the primary antibody incubation. Plates were then washed four times with PBST, and the ELISA was performed as above. The avidity index of the SD1 antibodies was calculated using a previously reported method.^61^

Peptide libraries for the antigens PvDBPII and DEKnull-4 were screened by ELISA performed as described above. Each peptide from the library was tested individually in duplicate. To account for plate-to-plate variation, we included a control well coated with peptide L2 (MKNSNYKRKRRERDWDSNTK) at 1.0 μg/mL and tested against the monoclonal antibody 3D10 at 0.008 μg/mL. ODs were converted to arbitrary units (AU) relative to the L2 control well that was run on each plate, according to the formula: AU = [(OD_Lib-peptide_ – Background)/(OD_L3-well_– Background)]∗100. Background was the OD of the peptide-coated well with secondary antibody alone.

#### Preparation of SD1p-specific B cell tetramers

To stain B cells specific to SD1p we used tetramers based on the biotinylated SD1p peptide described in the key resources table. Biotinylated SD1p was conjugated to phycoerythrin (PE) or allophycocyanin (APC). The tetramers were generated by mixing 8.34 nmol of biotin-SD1p with 1.668 nmol of streptavidin-conjugated PE or streptavidin-conjugated APC in a total volume of 100 μL, 5:1 molar ratio. Tetramers were incubated for a minimum of 72 h at 4°C in dark before use.

#### Lymphocyte isolation and flow cytometry

The lymph nodes were mashed and filtered through a 70 μm cell strainer in flow buffer (HBSS containing 2% FBS). Cells were then washed and resuspended with flow buffer. Fc-receptors were blocked with 10 μg/mL TruStain fcX antibodies incubating for 10 min at 4°C and washed once with flow buffer. Cells were then incubated with the antibody master mix and tetramer at a final concentration of 1 μg/mL for 30 min in the dark at 4°C. Cells were washed twice prior to staining with 1% 7AAD to identify live cells. Results were obtained using a Fortessa-SORP flow cytometer, and data were analyzed using FlowJo software (version 10.10). GC B cells were selected as B220+ CD19^+^ IgD-GL7+ live single cells.

### Quantification and statistical analysis

Data analyses were performed using GraphPad PRISM 10 software (Inc., CA, San Diego, CA, USA). The Kruskal-Wallis test with post hoc Dunn’s multiple-comparison test was applied to compare IgG responses between adjuvants or antigens. two-way ANOVA with *post hoc* Tukey’s, Dunnet’s or Šídák’s multiple comparison was used to compare IgG titers or B cell frequency between different mouse strains, antigens and adjuvants. The two-tailed Mann–Whitney U-test was used to compare continuous variables in two groups. *p* values <0.05 were considered significant.
