# Supplementary material for: Adjuvants and MHCII modulate the immunogenicity of subdominant epitopes in Plasmodium vivax Duffy binding protein
Source: iScience. 2025 Sep 23;28(10):113630. doi: 10.1016/j.isci.2025.113630 (PMC12547815; doi:10.1016/j.isci.2025.113630)
Supplement: Document S1. Figures S1–S7 [file mmc1.pdf]

iScience, Volume *28*

Supplemental information

Adjuvants and MHCII modulate the immunogenicity of subdominant epitopes in *Plasmodium vivax* Duffy binding protein

Daniel Ferrer Vinals, Mohammad Rafiul Hoque, Opeyemi Ernest Oludada, Ethan B. Jansen, Catherine J. Mitran, Jhon R. Enterina, Matthew S. Macauley, Michael T. Hawkes, and Stephanie K. Yanow

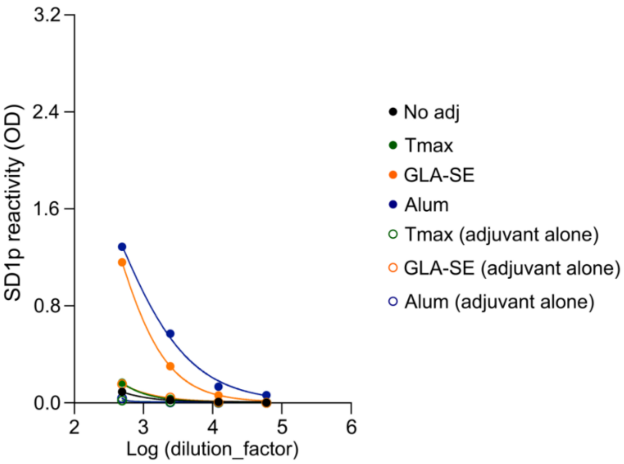

Supplementary Fig. 1. Titration curves of the IgG responses against SD1p. BALB/c mice were immunized subcutaneously with recombinant PvDBPII in the absence of adjuvant or with Tmax, GLA-SE or alum. Open circles represent the control groups where BALB/c mice were immunized with each adjuvant alone. Titration curves were fitted to the data using a sigmoidal function, with the log dilution on the x-axis and the corresponding OD value on the y-axis.

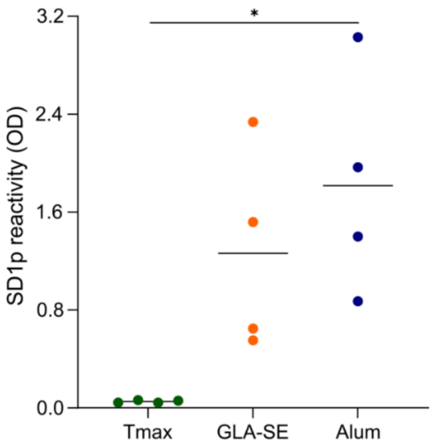

Supplementary Fig. 2. GLA-SE and alum increased the immunogenicity of SD1. Male BALB/c mice were immunized subcutaneously with recombinant PvDBPII adjuvanted with Tmax, GLA-SE or alum (n = 4 per group). IgG responses against SD1p were measured on day 45 at 1/500 dilution, with background from adjuvant alone subtracted from each group. Statistical analysis was performed using Kruskal-Wallis test (P = 0.0066) with *post hoc* Dunn's multiple comparison test. *$P < 0.05$. Solid lines represent the mean.

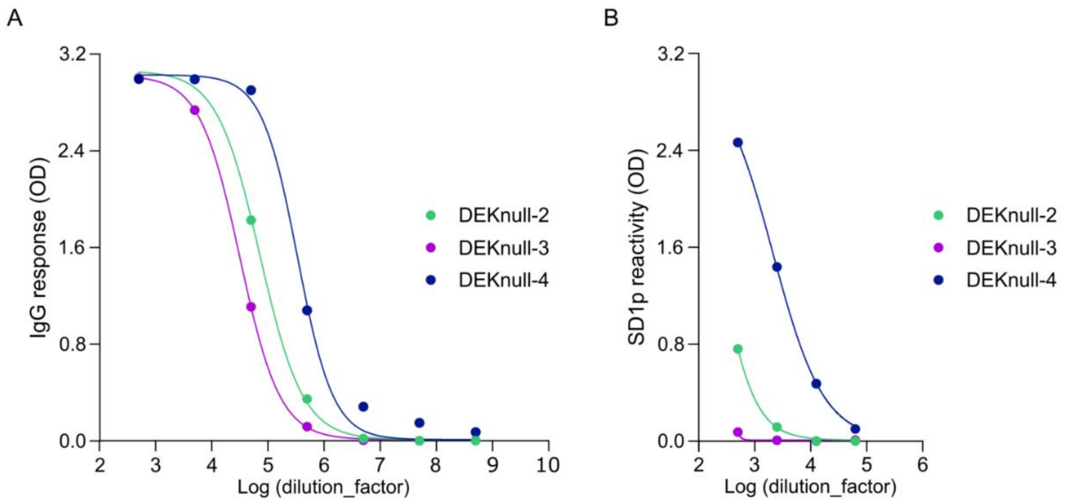

Supplementary Fig. 3. Titration curves of the IgG responses from BALB/c mice immunized with PvDBP mutants. A) The immunogen-specific IgG responses following immunization with recombinant DEKnull-2 (green), DEKnull-3 (purple), and DEKnull-4 (blue), adjuvanted with alum. B) The corresponding SD1p-specific IgG responses for each immunization group. Titration curves were fitted to the data using a sigmoidal function, with the log dilution on the x-axis and the corresponding OD value on the y-axis.

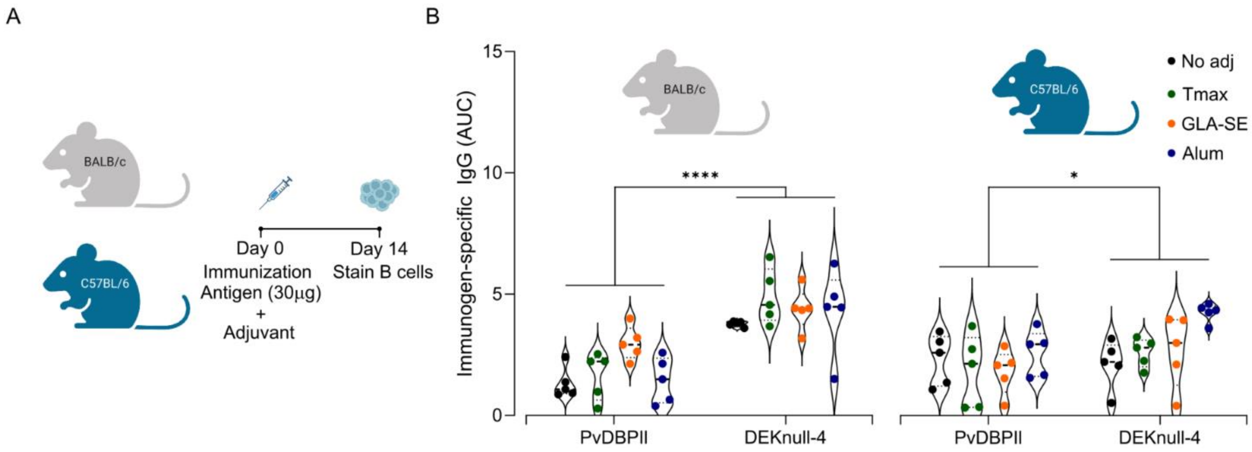

Supplementary Fig. 4. Immunogen-specific IgG responses at day 14 after immunization. A) Experimental set up. BALB/c and C57BL/6 mice were immunized subcutaneously with a single dose of 30 μg of recombinant PvDBPII or DEKnull-4 alone or adjuvanted with Tmax, GLA-SE or alum (n = 5 per group). B) Immunogen-specific IgG responses were measured on day 14 post-immunization. Data are presented as AUC. Dashed lines represent the median and stippled lines represent the quartiles. Statistical analysis was performed using two-way ANOVA with *post hoc* Tukey's multiple comparison test. For BALB/c, $P_{\text{adjuvant}} = 0.0910$; $P_{\text{antigen}} < 0.0001$ and for C57BL/6, $P_{\text{adjuvant}} = 0.0371$; $P_{\text{antigen}} = 0.0265$. *$P < 0.05$, ****$P < 0.0001$.

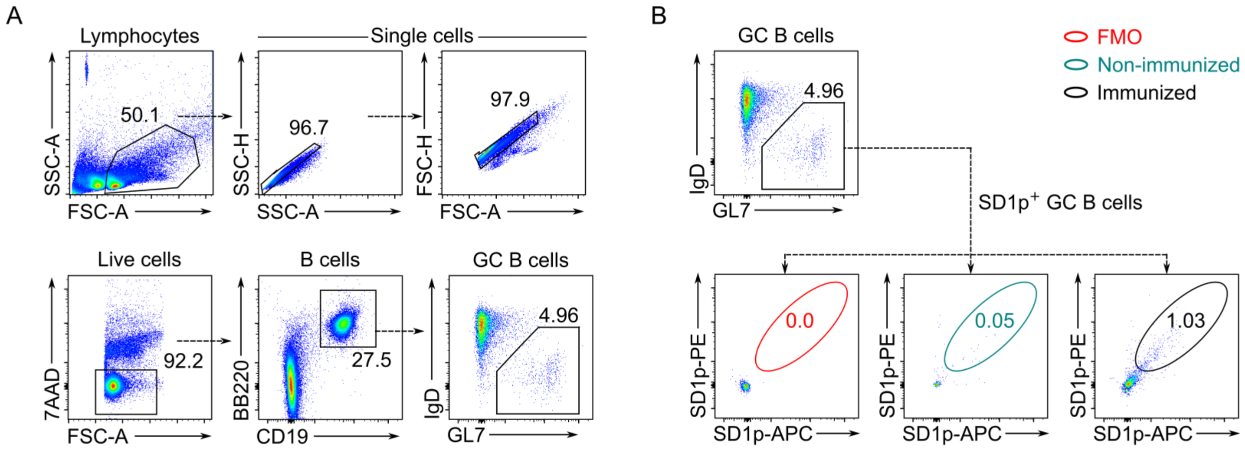

Supplementary Fig. 5. Flow cytometry gating strategy to analyse SD1p$^+$ GC B cells. A) Lymph node cells were identified by forward scatter area (FSC-A) vs side scatter area (SSC-A) density plots. Single cells were gated on relative SSC-A vs side scatter height (SSC-H) and relative FSC-A vs forward scatter height (FSC-H). Live cells were identified based on the 7AAD viability dye. B cells were gated as CD19+ and B220+. From the B cell population, GC B cells were defined as IgD- and GL7+ cells. B) From the GC B cells, double positive cells for streptavidin APC and streptavidin PE tetramers were identified as SD1p$^+$ GC B cells using non-immunized animal (pale green) and FMO (red) to discard non-specific staining.

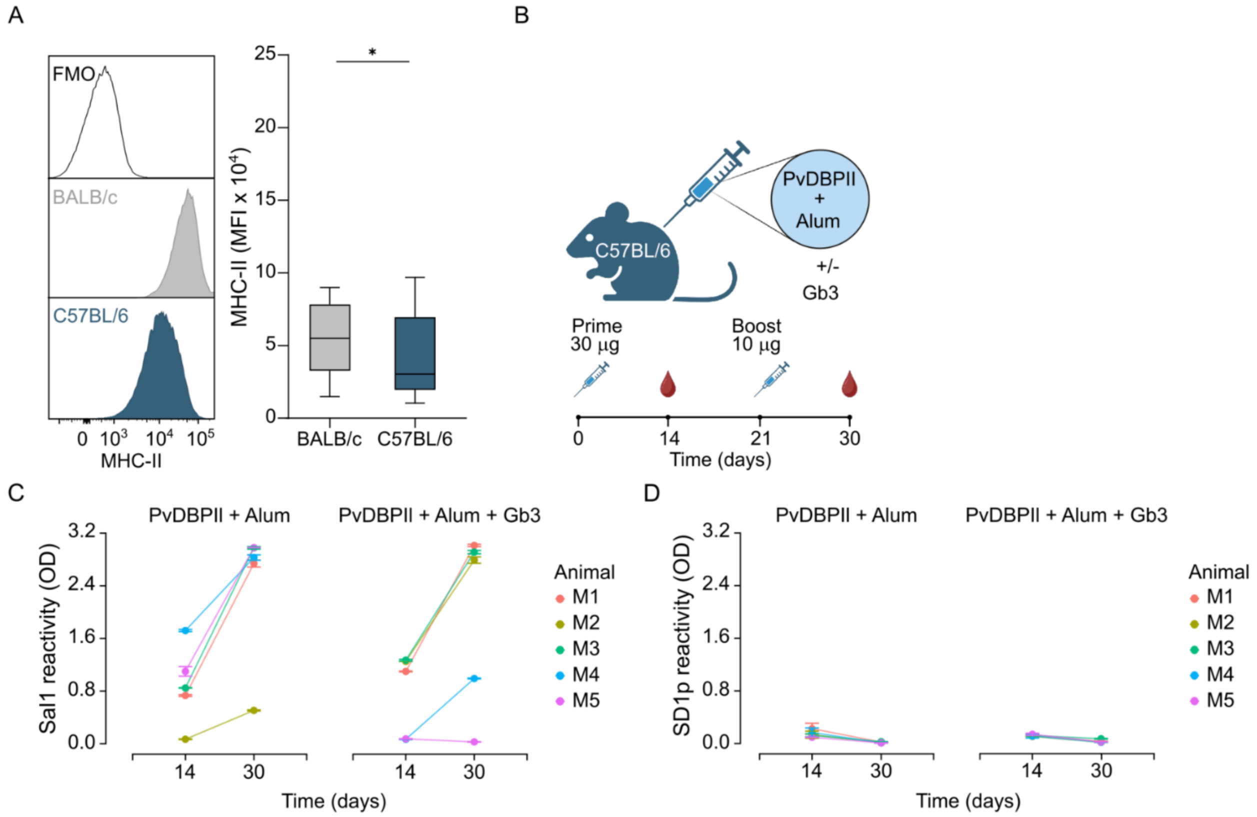

Supplementary Fig. 6. Gb3 did not overcome the subdominance of SD1 in C57BL/6 mice. A) Quantification of MHC-II expression on SD1p-specific B cells with histogram overlays showing the MHC-II staining on the two mouse strains BALB/c (n = 40) and C57BL/6 (n = 40) compared to the unstained population (FMO). Solid lines on data plots represent the median. B) Immunization scheme for Gb3 experiment. C) Effect of the Gb3 ganglioside on the IgG responses against PvDBPII (1/2500) elicited in C57BL/6 mice (n = 5 per group) immunized with PvDBPII and adjuvanted with alum. Responses were measured on days 14 and 30. D) Effect of Gb3 on anti-SD1p antibodies (1/500). For A, results from 40 mice per group were compared using a two-tailed Mann-Whitney U test ($P = 0.0107$). $^*P < 0.05$.

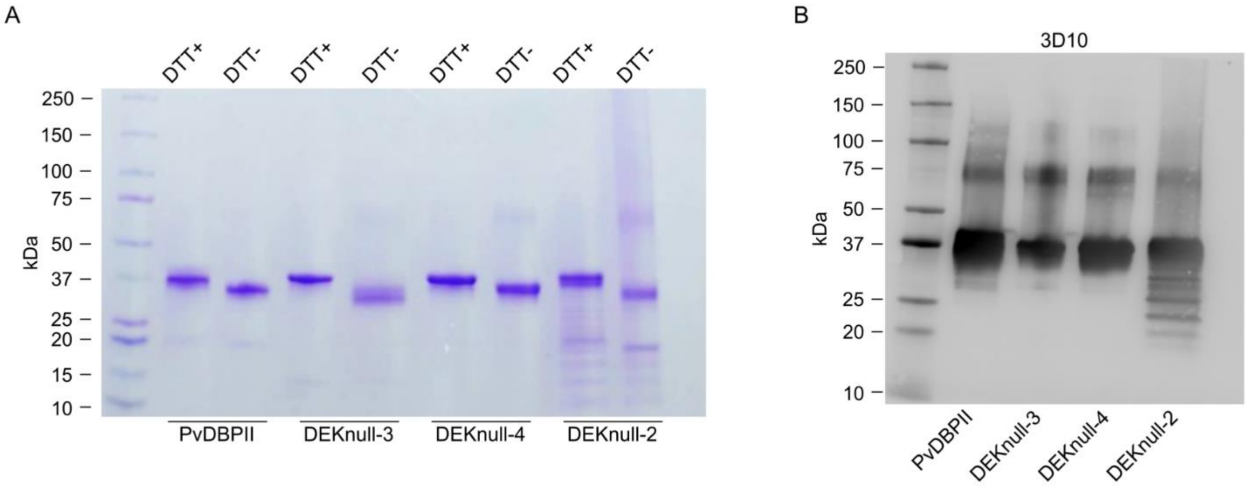

Supplementary Fig. 7. Production of recombinant antigens. A) SDS-PAGE with the recombinant proteins PvDBPII, DEKnull-2, DEKnull-3, and DEKnull-4 stained with Coomassie blue. The differential mobility of the proteins under reducing conditions was analyzed by the incubation of the samples with (+) or without (-) DTT. B) Western blot analysis of the recombinant proteins showing the recognition by the monoclonal antibody 3D10.
